# Review on drug repositioning of some FDA-approved drugs for treatment of cancer/microbial diseases

**DOI:** 10.1039/d6ra03396b

**Published:** 2026-07-03

**Authors:** Magda M. F. Ismail, Taghreed Z. Shawer, Omneya M. ElBakry, Eman A. A. Baumy, Sally I. Eissa

**Affiliations:** a Department of Pharmaceutical Medicinal Chemistry and Drug Design, Faculty of Pharmacy (Girls), Al-Azhar University Cairo 11754 Egypt m.elalfy101@gmail.com magdaismail@azhar.edu.eg

## Abstract

This approach drastically cuts development times and expenses by utilizing pre-existing safety and pharmacokinetic data. It works very well to fulfill unmet needs in infectious diseases, uncommon diseases, and cancer. Here, we provide an overview of FDA-approved medications that have been repurposed, covering biological features, modes of action, and synthetic processes.

## Abstract 2

Drug repurposing, also known as repositioning, is a strategic pharmaceutical method that provides a quicker and more economical substitute for *de novo* drug discovery by finding new therapeutic uses for licensed, unsuccessful, medications.

## Graphical abstract

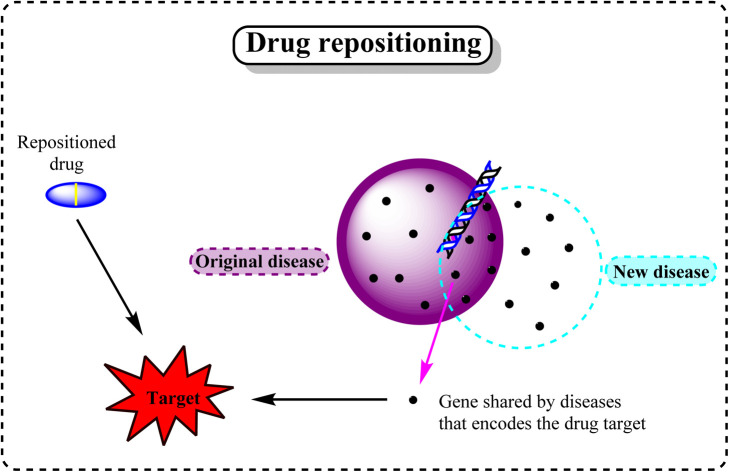

## 1 Introduction

Drug repurposing, or repositioning, is the process of identifying new therapeutic uses for current, approved, or failed medications, offering a speedier and more cost-effective alternative to traditional drug development. This approach is crucial for meeting urgent medical demands since it minimizes development risk and time by using chemicals with proven safety profiles.^1–3^ Finding novel therapeutic applications for medications that have already received approval or are in advanced stages of clinical development is the ultimate goal of therapeutic drug repurposing, which aims to promote substantial advancements in pharmacotherapy. In an era of high-throughput drug screening, which nevertheless has a not insignificant failure rate, drug repurposing offers a logical and alluring alternative strategy as a tool to find successful therapeutic uses for known pharmaceuticals.^4^ A medicine is more amenable to repositioning the more successfully it can be repurposed.^5^ These surveys may reveal some biological characteristics of medications that are already known.^6^ There is a case to be made for accelerating access to known marketed compounds due to high attrition rates, long-term dedication to ethical and regulatory difficulties, and the cost-effectiveness of new chemical entities avoiding the drawn-out discovery process. About 30% of new FDA-approved medications and vaccines have been successfully repurposed in recent years. The current state of pharmaceutical repurposing techniques for a range of illnesses is the main topic of this study. There have been attempts to provide the synthesis, applications, and mechanisms of action of pharmaceuticals.

Traditional methods for drug repurposing were due to serendipity or the unintended concurrence of a drug and a disease.^7^ With advances in computational drug discovery, several innovative strategies have been developed that can facilitate drug repurposing. The most common methods are ligand-based and structure-based drug design approaches.^8^ The ligand-based methods of drug discovery are based on the similarity of compounds, which can be determined either by comparing their structural fingerprints or by calculating the similarity of pharmacophores.^9^ This approach is not very successful if the molecules to be compared are flexible, large, and dissimilar scaffolds.^10^ In structure-based drug design, molecular recognition is a consequence of the molecule's ability to adopt the proper conformation in relation to a target.^11^ This method is less computationally expensive and time-consuming than the ligand-based method of design.^12^ Conceptually, both approaches require a degree of prior knowledge of the target before initiating any drug design.^13^

### 1.1 Rational for repurposing drugs

Drugs rarely bind to a particular target. A medication's “off-target” or secondary effects can often be employed to treat an entirely unrelated ailment. Pre-approved drugs have completed comprehensive phase I clinical testing, thus their toxicity, metabolic routes, and human side-effect profiles are well-documented. Avoiding early-stage development also reduces costs and research and development (R&D) schedules by half, unlike *de novo* drug discovery, which affects speed and cost effectiveness. As a result, even when two drugs have comparable mechanisms of action, their repurposing outcomes can vary greatly for the following reasons: various target subtypes, dosage and toxicity thresholds, off-target effects, pharmacokinetics (PK), and bioavailability.^3^

The mechanisms of action, synthetic pathways, and therapeutic applications of FDA-approved pharmaceuticals in the US, as well as their use following repurposing, will all be covered in this overview.

### 1.2 Repurposed drugs for cancer therapy

#### 1.2.1 Aspirin

In 1899/1900, Bayer developed and patented acetyl salicylic acid for use in treating pain and inflammation. It is perhaps arguably the most commonly used medication in human history.^14–16^ It was later shown that this medication preferentially inhibited cyclooxygenase after significantly inhibiting prostaglandin production.^17,18^ Furthermore, it has been shown that cyclooxygenase catalyzes the transformation of arachidonic acid into thromboxanes and prostaglandins.^19^ Pain and fever are caused by these two substances, which are released during inflammation. The strong affinity and selectivity of acetylsalicylic acid for cyclooxygenase can be explained by structure–activity relationship studies, which have demonstrated that the inhibitor's acetyl group is securely linked inside the enzyme structure.^20^ Strong, long-lasting inhibitory activity is caused by this selective, irreversible acetylation of serine at the enzyme's active region.^21^

Additionally, aspirin has been reprofiled for prostate, breast, and colorectal cancer, among other cancer types.^22^ Recent years have seen increased evaluation of the relationships between aspirin use and chemotherapy medications. Aspirin has been shown to boost doxorubicin's anticancer activity in breast cancer.^23^ Aspirin and doxorubicin together prevented MCF-7 breast cancer cells from proliferating and caused apoptosis.^24^ Additionally, *in vitro* experiments shown that aspirin could enhance breast cancer cells' susceptibility to doxorubicin by blocking the MEK/ERK signaling pathway.^25^ As a result, the combination of both medications changes the potential of the mitochondrial membrane, which causes an increase in intracytoplasmic calcium, the release of cytochrome C and AIF proteins, and the triggering of caspase-induced death.^26^ Additionally, the FDA authorized its use in 1985 for the prevention of acute myocardial infarction.

##### 1.2.1.1 Synthesis

**Fig. 1:**
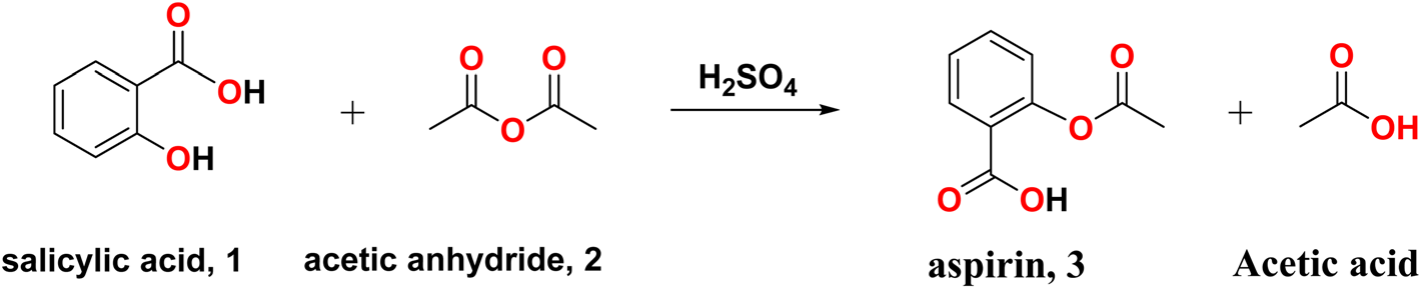
Synthesis of aspirin.

A determined amount of salicylic acid, 1 is mixed with excess acetic anhydride, 2 while sulfuric acid is present as a catalyst. Acetic acid and acetylsalicylic acid 3 are produced by heating the combination. Water is introduced to the process to eliminate any remaining acetic anhydride,^27^ (Fig. 1).

#### 1.2.2 Thalidomide

Thalidomide, also known as Thalomid, was approved by the FDA in 1998 to treat severe leprosy (erythema nodosum leprosum, ENL). Unfortunately, thousands of children experienced limb malformations due to its severe teratogenic side effects.^28^

In 2006, thalidomide was repositioned and authorized in combination with dexamethasone for the treatment of multiple myeloma, a blood cancer. High-dose intravenous (IV) ascorbic acid is investigated for its potential to selectively induce reactive oxygen species (ROS) and target tumor microenvironments. Additionally, thalidomide works by binding to the protein cereblon (CRBN) and acting as a “molecular glue” to force it to ubiquitinate and degrade certain proteins (like Ikaros and Aiolos), changing the function of immune cells, inhibiting inflammatory cytokines like TNF-α, and promoting anti-angiogenesis (blocking new blood vessels).^29^ Furthermore, thalidomide was used to treat cancer after its anti-angiogenic qualities were discovered. Because the angiogenic process that promotes tumor growth and chronic inflammation are similar, thalidomide may have an anti-inflammatory impact in cancer therapy. The anti-TNF-α effects of thalidomide are important because TNF-α contributes to angiogenesis by raising the expression of crucial endothelium integrin.^30^

##### 1.2.2.1 Synthesis

**Fig. 2:**
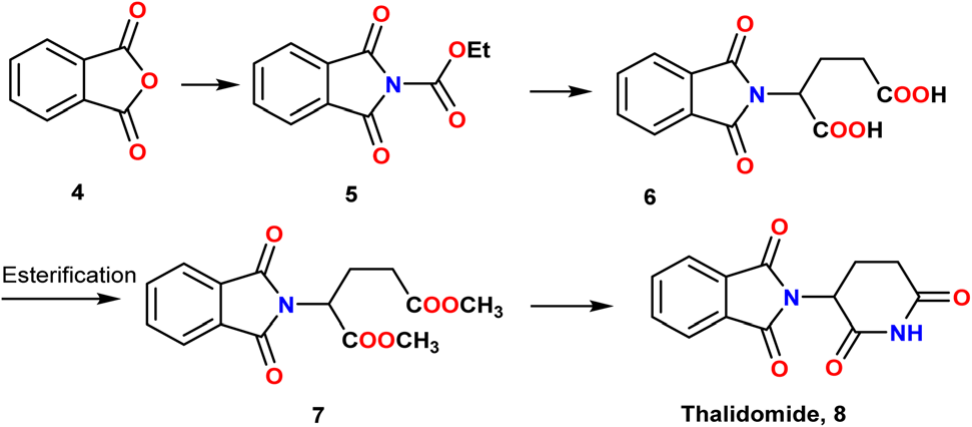
Synthesis of thalidomide.

In its production, phthalic anhydride 4 is activated to ethyl 1,3-dioxoisoindoline-2-carboxylate, 5 which is reacted with l-glutamic acid to make *N*-phthaloyl-dl-glutamic acid, 6. Then 6 underwent esterification to yield the matching ester, 7. Afterwards, compound 7 underwent cyclization in presence of ammonium acetate and diphenyl ether to form thalidomide 8,^31^ (Fig. 2).

#### 1.2.3 Ascorbic acid

The human diet has been regularly fortified with ascorbic acid, also known as vitamin C, which is an essential component. 1947 marks the “Initial U.S. Approval” date found on many official FDA labels for ascorbic acid. Because the human body cannot produce it, it is a necessary vitamin for living. An adequate intake of ascorbic acid, which is necessary for the synthesis of connective tissue, the creation of blood clots, catecholamines, and the antioxidation of vitamin E, is absolutely necessary for the growth and development of the human body. Additionally, it serves as a reducing agent in the citric acid cycle, aids in the absorption of iron from the diet, and plays a significant role in the synthesis of carnitine, steroids, and neuropeptides. It is essential for several metabolic processes, including brain formation and neurotransmission.^32^

One efficient and intriguing combo option to target the tumor micro-environment (TME) is repurposing Vit C. Although vitamin C is known for its health benefits, it can also be reprofiled as a powerful agent that kills cancer cells based on three factors: (1) whether the antioxidant qualities of vitamin C encourage tumor diffusion; (2) whether vitamin C could be used in conjunction with dietary therapies like fasting and ketogenesis; and (3) the impact of vitamin C on the microbiome and bacterial-derived vitamin C on efficacy.^33^

##### 1.2.3.1 Synthesis

**Fig. 3:**
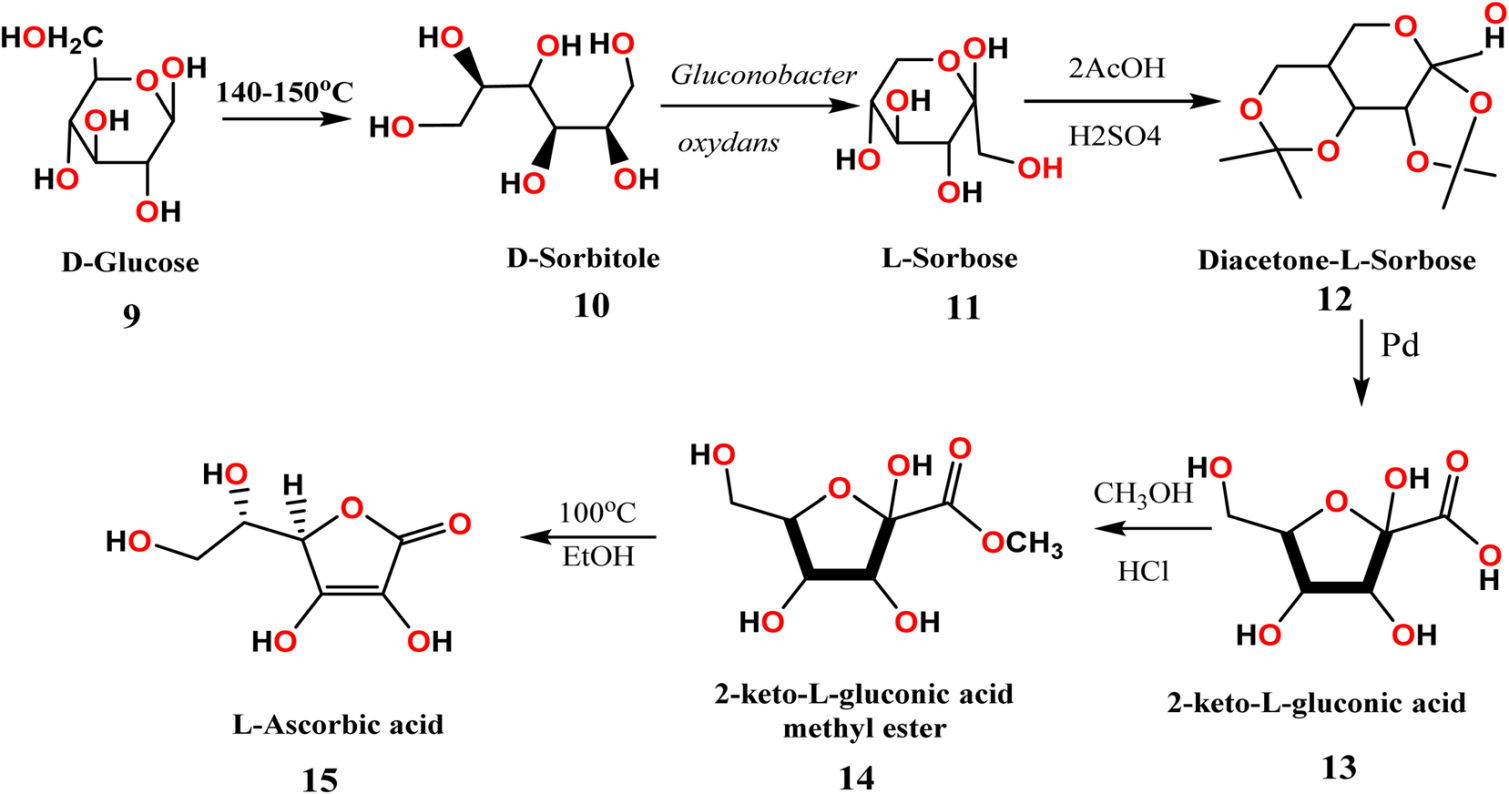
Synthesis of ascorbic acid.

The Reichstein–Grüssner process, created in 1933, was the main industrial approach until recently. Heating d-glucose 9 to 140–150 °C with high pressure using nickel catalyst yields d-sorbitol 10, which afforded l-sorbose, 11 through a bio-oxidation with *Gluconobacter oxydans*. Then, protection of hydroxyl groups of 11 with acetone and sulfuric acid afforded the tricyclic derivative (diacetone-l-sorbose) 12. The remaining hydroxyl group is next oxidized to carboxylic acid using a palladium catalyst, producing 2-keto-l-gluconic acid (2KGA), 13 as an essential intermediary. Then, the latter was esterified by treatment with HCl and methanol to form 2-keto-l-gluconic acid methyl ester 14. Finally, the ester was then heated at 100 °C in ethanol to trigger lactonization and enolization to form l-ascorbic acid 15,^34^ (Fig. 3).

#### 1.2.4 Metformin

Metformin, the first-line therapy for Type 2 Diabetes (T2DM), was approved in the UK in 1957. Metformin lowers blood glucose, particularly in type 2 diabetes, by decreasing intestinal glucose absorption, hepatic glucose production (gluconeogenesis), and enhancing insulin sensitivity to improve peripheral glucose uptake and utilization.

Because metformin is involved in both type 2 diabetes and cancer, it is being intensively investigated for potential repurposing. Metformin is being researched extensively for use as a treatment for neurological or cardiovascular disorders, an anti-aging and life-extending medication, and an anti-cancer agent. Its potential use as an anticancer drug has drawn attention due to its capacity to inhibit the mTOR pathway and activate AMPK. Metformin also stops the generation of cellular energy, which is necessary for the growth of cancer, by inhibiting mitochondrial complex I.^35^ Metformin demonstrated the potential to significantly improve cardiovascular health by lowering aldosterone-induced heart damage, lowering inflammation and oxidative stress, and improving lipid profiles, in addition to its core role in managing diabetes.^36^ Moreover, metformin is used off-label to control weight in insulin-resistant, non-diabetic people by reducing fat storage, improving insulin sensitivity, preventing hepatic gluconeogenesis, and boosting fat-burning (AMPK pathway). Small but noticeable weight loss is often seen in this people.^37^ Metformin is the recommended globally due to its well-established and good safety profile.^38^

##### 1.2.4.1 Synthesis^39^

**Fig. 4:**
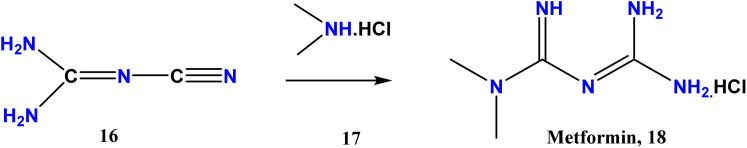
Synthesis of metformin.

Microwave-assisted synthesis of metformin hydrochloride (18), a biguanide antidiabetic medication, is created by nucleophilically adding dimethylamine (derived from its hydrochloride salt), 17 to the nitrile group of 2-cyanoguanidine (dicyandiamide), 16. This process precipitates metformin with a yield of 96%, (Fig. 4).

#### 1.2.5 Niclosamide

Niclosamide (NIC), a protonophore that uncouples oxidative phosphorylation in helminth mitochondria, is an FDA-approved oral anthelmintic medication that efficiently treats tapeworm infections in 1982. Through this mechanism, the parasite's mitochondrial membrane potential is disrupted, ATP generation is inhibited, and glucose uptake is blocked, ultimately starving and killing the worm. Because of its restricted absorption, which usually confines detrimental side effects to the gastrointestinal system, the drug is most suited for treating enteric infections.^40^

In recent years, NIC has shown therapeutic potential and multifunctional pharmacological activity. Repurposing NIC as anti-cancer, metabolic regulatory, immunotherapeutic, antiviral, and antibacterial agents is one of these.^41,42^ Numerous biological processes and signaling pathways, such as the Wnt/β-catenin, mTOR, STAT3, NF-κB,^43–47^ and Notch signaling pathways,^48^ have also been demonstrated to be regulated by it. It was also discovered that NIC stopped non-breast cancer stem cells from developing into cancer stem cells. It was demonstrated that NIC reduces mTORC1 signaling and does so without the assistance of Akt, ERK, or AMK.^49^ Niclosamide has been shown to have anti-cancer properties in human breast, prostate, colon, ovarian, multiple myeloma, acute myelogenous leukemia, glioblastoma, head and neck, and lung cancer cells. Niclosamide can cause cancer cell cycle arrest, growth inhibition, and apoptotic death in a variety of cancer types by blocking the several signaling pathways that control cancer start and development.^50^ It increased the anti-proliferative effect of oxaliplatin, a medication frequently used to treat colorectal cancer (Caco2 cells) and colorectal cancer explant cells.^51^

##### 1.2.5.1 Synthesis^52^

**Fig. 5:**
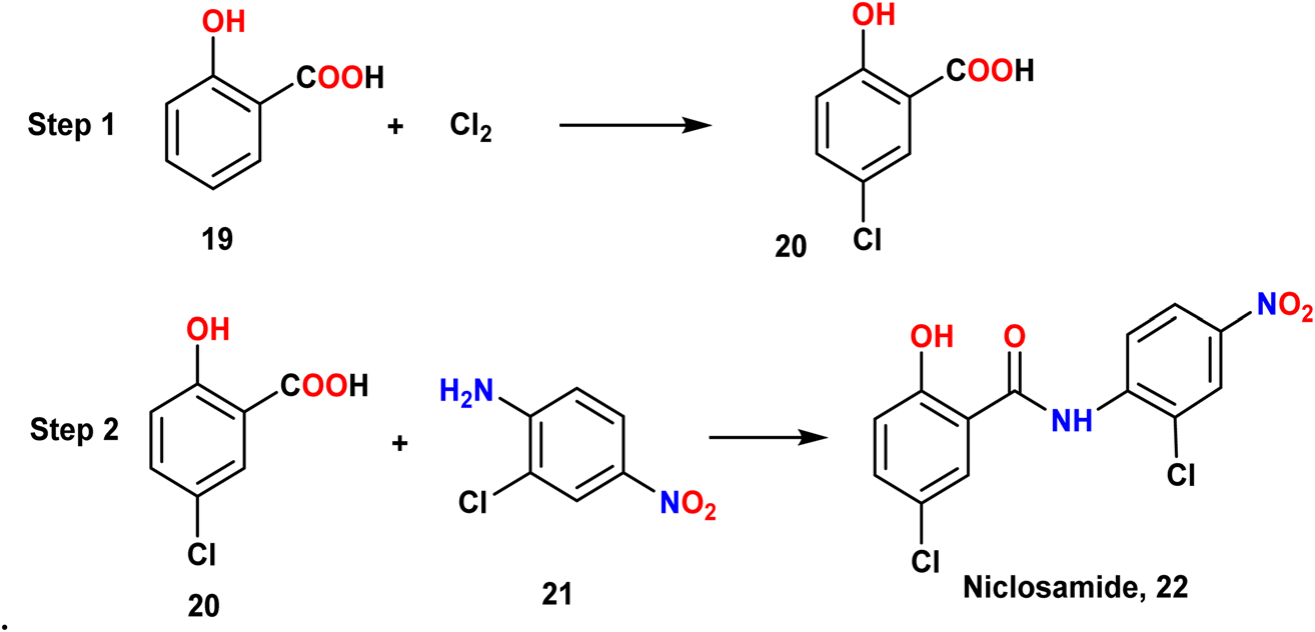
Synthesis of niclosamide.

Niclosamide is made industrially by chlorinating salicylic acid 19 in chlorobenzene to create 5-chlorosalicylic acid, 20. Next, 5-chlorosalicylic acid 20 and 2-chloro-4-nitro-aniline 21 are condensed to form niclosamide 22, (Fig. 5).

#### 1.2.6 Gemcitabine

A nucleoside analog used extensively in oncology, received its initial FDA approval in the mid-1990s. It is an antiviral medication that has been shown to prevent infection of many DNA or RNA viruses by permanently blocking the DNA polymerase enzyme as its nucleoside counterpart (deoxycytidine).^53^ Gemcitabine can be transformed into its corresponding triphosphates so that it can be integrated into both host DNA and RNA. Gemcitabine's short-lived, self-limited, and rarely irreversible hepatotoxicity might result in fatal liver necrosis or cholestatic failure. In order to detect significant injury, it is recommended to routinely monitor liver function tests (LFTs). The goal of repurposing gemcitabine is to increase its effectiveness against solid tumors, particularly pancreatic cancer, and overcome resistance by combining it with other medications or altering it to lessen systemic toxicity. Its short half-life and quick inactivation can be addressed by employing statins, antiviral medications, or cell-penetrating peptides to increase its anticancer effects. As an antimetabolite (an analogue of deoxycytidine), gemcitabine permanently prevents further DNA synthesis.^54^

##### 1.2.6.1 Synthesis^55,56^

**Fig. 6:**
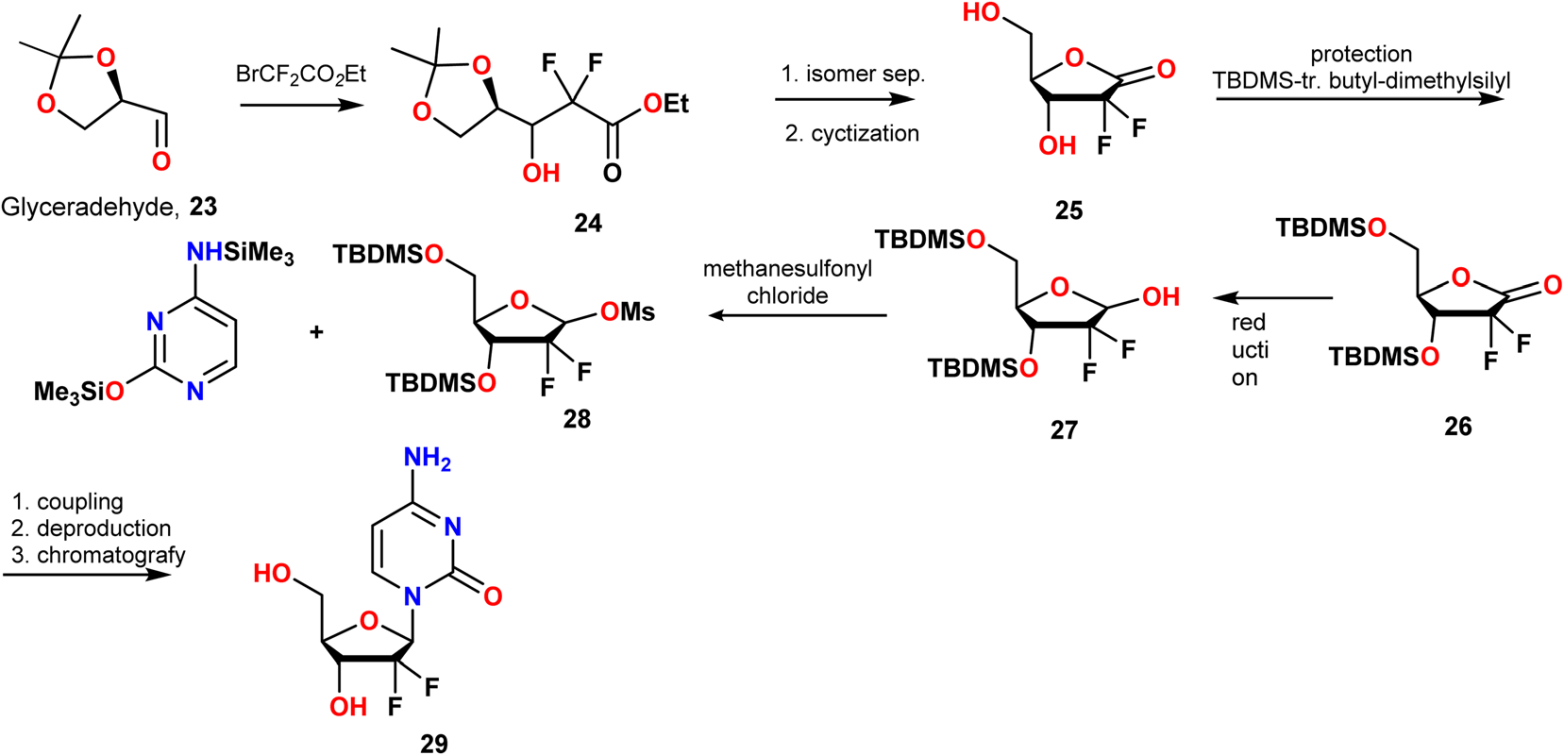
Synthesis of gemcitabine.

Its synthesis begins with the addition of fluorine to compound 23 using bromodifluoro-acetate ethyl bromodifluoroacetate, activated Zn, and THF/diethyl ether (1 : 1) to produce compound 24, followed by cyclization using Dowex-50W-X12 H+ resin and MeOH (a strongly acidic ion-exchange resin) to furnish 25. Then, protection for alcohol groups using *tert*-butyldimethylsilyl trifluromethanesulfonate (TBDMS), 2,6-lutidine, and DCM to give compound 26. Afterwards, compound 27 is produced by a reduction process with DIBAL-H and toluene, whereas compound 28 is produced by adding methanesulfonyl chloride in NEt3, and DCM to the hydroxyl derivative, 27. Lastly, nuclobase is added by refluxing TMSOTF in dichloroethane with silylated cytidine 29, (Fig. 6).

#### 1.2.7 Orlistat

Orlistat is an anti-obesity drug that was approved by the FDA in 1999 (DR_XY40428). It was first used to reduce weight by reversibly inhibiting pancreatic and gastric lipases, which are important enzymes for breaking down dietary fat. By attaching to their active sites, orlistat stops triglyceride hydrolysis and reduces fat absorption by roughly 30%.^57^ multivitamin is advised since it can cause gastrointestinal problems such diarrhea, hepatotoxicity, osteoporosis, and interfere with the absorption of fat-soluble vitamins.^58^

Orlistat's capacity to function as a Fatty Acid Synthase (FASN) inhibitor makes it a prospective therapeutic candidate in oncology because many quickly proliferating cancer cells depend on the overproduction of fatty acids. However, orlistat has recently been repurposed as an anti-tumor agent. It may result in the death or apoptosis of tumor cells, disrupt the regulation of tumor cell cycles, inhibit fatty acid synthase activity (an enzyme essential for tumor cell proliferation leading to tumor cell apoptosis), enhance ferroptosis, prevent tumor angiogenesis, and improve tumor cells' glycolytic activity.^59^

##### 1.2.7.1 Synthesis^60^

**Fig. 7:**
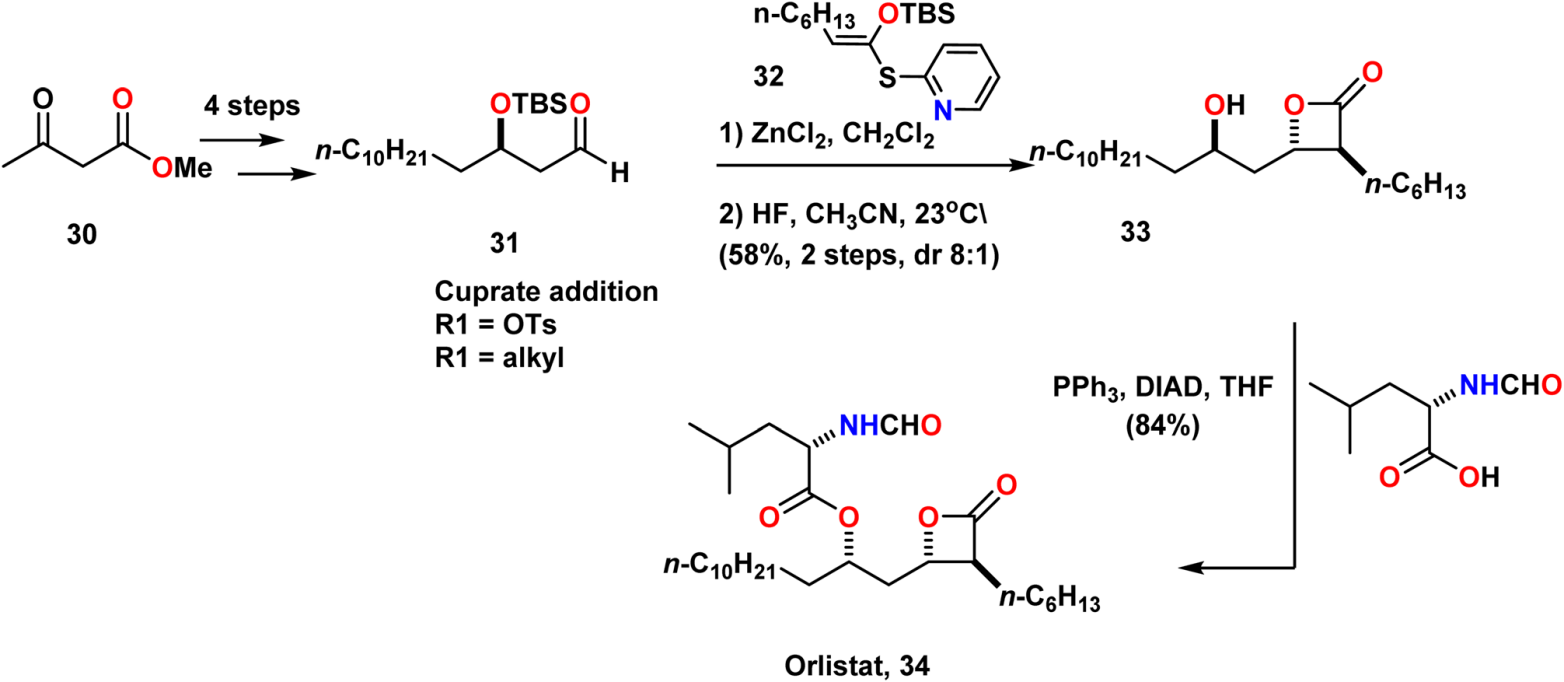
Synthesis of orlistat.

Methyl acetoacetate 30 was alkylated with crotyl bromide (Acros; mixture of *E*/*Z* isomers), followed by Noyori reduction, alcohol protection with TBSCl, and ester reduction with DIBAl-H to create aldehyde 31. Next, TMAL reaction with ZnCl_2_ which was fused and allowed to cool to room temperature under a nitrogen flow; followed by addition a solution of the aldehyde 31 in 2.5 mL of CH_2_Cl_2_ and ketene acetal, 32 to afford the corresponding b-lactone 33. Orlistat 34 is made from b-lactone 33, triphenylphosphine, *N*-formyl-l-leucine, and DIAD in 2 mL of THF, (Fig. 7).

#### 1.2.8 Lidocaine

The well-known local anesthetic lidocaine (LIDO) was first approved in 1948 by FDA as lidocaine hydrochloride for medical use in the United States. It works by blocking sodium channels, which temporarily prevents the neurons in the local tissues where the drug is applied from communicating with the brain about feelings.^61^

Scientific data indicates that, it is being actively repurposed in clinical research as an anticancer agent and a systemic anti-inflammatory immunomodulator in addition to its conventional applications as a local anesthetic and a class Ib antiarrhythmic. Lidocaine may inhibit the growth of cancer cells both *in vitro* and *in vivo* through a variety of mechanisms, including the regulation of epigenetic modifications and the promotion of pro-apoptotic pathways, as well as the regulation of ABC transporters, metastasis, angiogenesis, and other processes. This information may be useful for the development of new drugs and for its continued use in cancer treatment. Furthermore, lidocaine is currently being tested in clinical settings to treat specific cancers.^62^

##### 1.2.8.1 Synthesis^63^

**Fig. 8:**
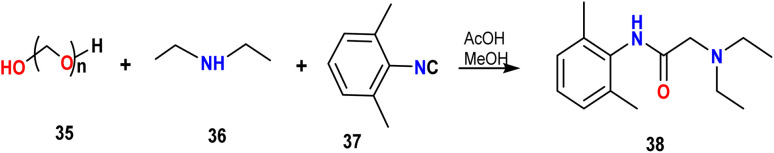
Synthesis of lidocaine.

Ugi three-component synthesis is used to create the topical anesthetic lidocaine 38 with exceptional atom economy. For a week, paraformaldehyde 35, diethylamine 36, and 2,6-dimethylphenyl isocyanide 37 were readily mixed in methanol with acetic acid at room temperature to furnish lidocaine 38, (Fig. 8).

#### 1.2.9 Losartan

Losartan, a common antihypertensive, was approved by the US Food and Drug Administration (FDA) in 1995 as an angiotensin receptor blocker (ARB) to treat several illnesses, such as diabetic nephropathy and hypertension. Losartan reduces thirst, cellular hypertrophy and hyperplasia, noradrenergic neurotransmission, angiotensin II-induced vasopressin release, adrenal catecholamine release, rapid and delayed pressor responses, and elevated sympathetic tone.^64^

However, it is being repurposed to alter tumor microenvironments because it inhibits the chemical processes (TGF-B) that cause tissue stiffness. It enhances the supply of oxygen and chemotherapy to solid tumors (such as pancreatic and breast malignancies) by decreasing collagen in tumors for its anti-fibrotic and anti-inflammatory properties, particularly targeting TGF-β signaling. Other areas of investigation include treating rare diseases like Osteogenesis Imperfecta (OI) and dystrophic epidermolysis bullosa (DEB), as well as tackling MRSA skin infections and fibrostenosing Crohn's disease. It decreased the amount of collagen I and the thickness of the muscularis propria, decreased inflammatory cell infiltrates, and restored villous structure. Additionally, losartan dramatically reduced the expression of interleukin-13, a cytokine linked to fibrosis, and profibrogenic genes (Col1a1, Mmp9, Igf1).^65^

##### 1.2.9.1 Synthesis^66^

**Fig. 9:**
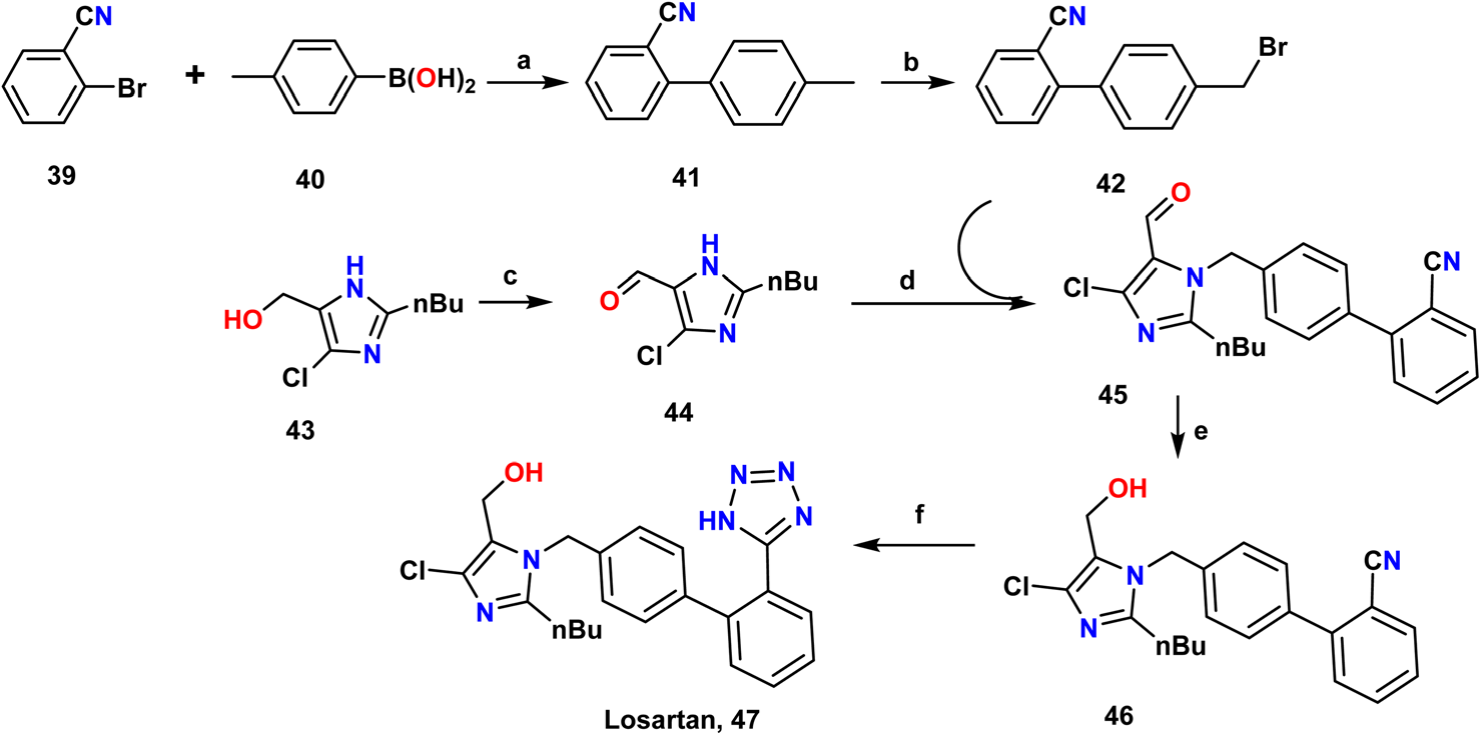
Synthesis of losartan. Conditions: (a) PdNPs, K_2_CO_3_, acetone/H_2_O (1 : 1), 35 °C, 24 h; (b) NBS, *hν*, ACN, 70 °C, 2 h; (c) TEMPO, iodine, toluene, aq. KHCO_3_, 20 °C; (d) K_2_CO_3_, EtOH, 45 °C, 24 h; (e) NaBH_4_, MeOH; (f) NaN_3_, ZnBr_2_, *n*-BuOH, 100 °C, 48 h.

The biphenyl scaffold 41 is produced by the Suzuki–Miyaura coupling of aryl halide 39 and aryl boronic acid 40. By brominating 41, the corresponding bromobenzyl derivative, 42, was produced. On the other hand, imidazole, 43 was oxidized to make imidazole carboxaldehyde, 44, which was then combined with the bromobiphenyl derivative, 42 to produce product 45*via* elimination of HBr molecule. The primary alcoholic group at p-5 of 46 was then restored by reducing the carboxaldehyde group in compound 45 with sodium borohydride. Finally, a tetrazole moiety is introduced in the synthesis of losartan 47 by a [2 + 3] cyclo-addition of azide and nitrile of compound 46, (Fig. 9).

#### 1.2.10 Statins

Strong lipid-lowering medications with additional pleiotropic effects are called statins. They can cure hypercholesterolemia because they inhibit 3-hydroxy-3-methylglutarylcoenzyme A (HMG-CoA) reductase. They are the most effective substances for lowering plasma cholesterol.^67^ Atorvastatin, one of the most widely prescribed statins for the management of hypercholesterolemia and the prevention of cardiovascular disease, received its first official approval from the FDA in the late 1996.^68^

According to a recent meta-analysis, the usage of statins repurposed as anticancer was significantly associated with a decrease in colorectal cancer-specific and total mortality. A meta-analysis of 59 073 patients with liver cancer revealed that statin use was substantially linked to a lower risk of hepatocellular carcinoma (HCC) progression when compared to non-statin users. The synthetic derivatives, fluvastatin, pitavastatin, cerivastatin, atorvastatin, and rosuvastatin share structural characteristics, such as fluoride side groups. Depending on their chemical structure, statins may have a variety of intracellular actions. Lipophilic statins, like atorvastatin, have a higher capacity to pass through cell membranes and enter hepatocytes and non-hepatocytes through passive diffusion than hydrophilic statins, such rosuvastatin and pravastatin. Additionally, compared to hydrophilic statins, lipophilic statins exhibit greater pro-apoptotic action. Lipophilic statins may be useful in the treatment of cancer because of their increased cytotoxic potential.^69^

##### 1.2.10.1 Synthesis^70^

**Fig. 10:**
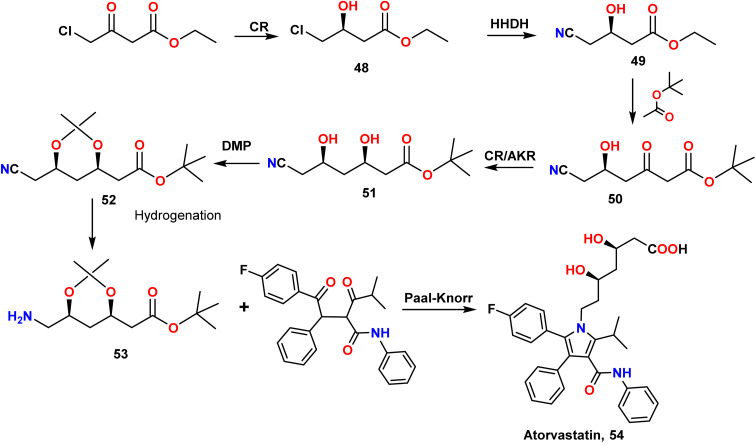
Synthesis of atorvastatin.

Carbonyl Reductase (CR) is used to bioreduce ethyl-4-chloro-acetoacetate to (*S*)-4-chloro-3-hydroxybutyric acid ethyl ester 48 during the synthesis of atorvastatin 54. (*S*)-4-chloro-3-hydroxybutyric acid ethyl ester 48 is subsequently biologically dehalogenated by halohydrin dehalogenase (HHDH) to form (*R*)-4-cyano-3-hydroxybutyric acid ethyl ester 49, which is the initial chiral center of the chiral diol unit. Following a Claisen ester condensation reaction, it is transformed into 6-cyano-(5*R*)-hydroxy-3-carbonylhexanoic acid *tert*-butyl ester, 50, which is subsequently reduced to 6-cyano-(3*R*, 5*R*)-*tert*-butyl dihydroxyhexanoate, 51. This is then reacted with 2,2-dimethoxypropane (DMP) to produce (4*R*, 6*R*)-6-cyanomethyl-2,2-dimethyl-1,3-dioxane-4-*tert*-butylacetate, 52 which is subsequently hydrogenated to (4*R*,6*R*)-6-aminoethyl-2,2-dimethyl-1,3-dioxane-4-*tert*-butylacetate, 53. The pyrrole ring of atorvastatin, 54 could be formed by a Paal–Knorr cyclocondensation of the highly substituted 1,4-diketone with primary amine of compound 53, (Fig. 10).

## 2 Repurposed drugs for microbial therapy

### 2.1 Tamoxifen

Tamoxifen (TAM) is a widely used selective estrogen receptor modulator (SERM) for treating and preventing estrogen receptor-positive (ER+) breast cancer. FDA first approved tamoxifen under the brand name Nolvadex in 1977 as a first-line hormone therapy for premenopausal women. It works by binding to estrogen receptors, acting as an antagonist in breast tissue to stop cancer cell growth, while acting as an agonist in other tissues.

However, because of its strong antibacterial, antifungal, and possibly anti-parasitic qualities, TAM is being repurposed. According to studies, it modulates autophagy and lysosomal activity to combat multidrug-resistant bacteria, tuberculosis, and heart problems associated with Duchenne Muscular Dystrophy (DMD). It works by disrupting bacterial membranes, killing fungi by interfering with calmodulin, and triggering apoptosis in parasites. TAM has demonstrated a direct inhibitory effect on the growth of *Mycobacterium tuberculosis* as well as strains of *Staphylococcus aureus* and methicillin-resistant*S. aureus* (MRSA).^71^ In a *Galleria mellonella* infection model, TAM was reported to decrease *Enterococcus faecium* growth and pathogenicity.^72^ Through the agonist activity of the G protein-coupled estrogen receptor,^73^ TAM was shown to have an indirect antibacterial effect by boosting the activity of immune cells represented by neutrophils against a variety of pathogenic bacteria, including MRSA, *Pseudomonas aeruginosa*, and *Escherichia coli*. When polymyxin B and TAM (tamoxifen) are combined, antibacterial activity is greatly increased, lowering minimum inhibitory concentrations (MICs) and regaining effectiveness against multidrug-resistant (MDR) Gram-negative bacteria.^74^

#### 2.1.1 Synthesis^75^

**Fig. 11:**
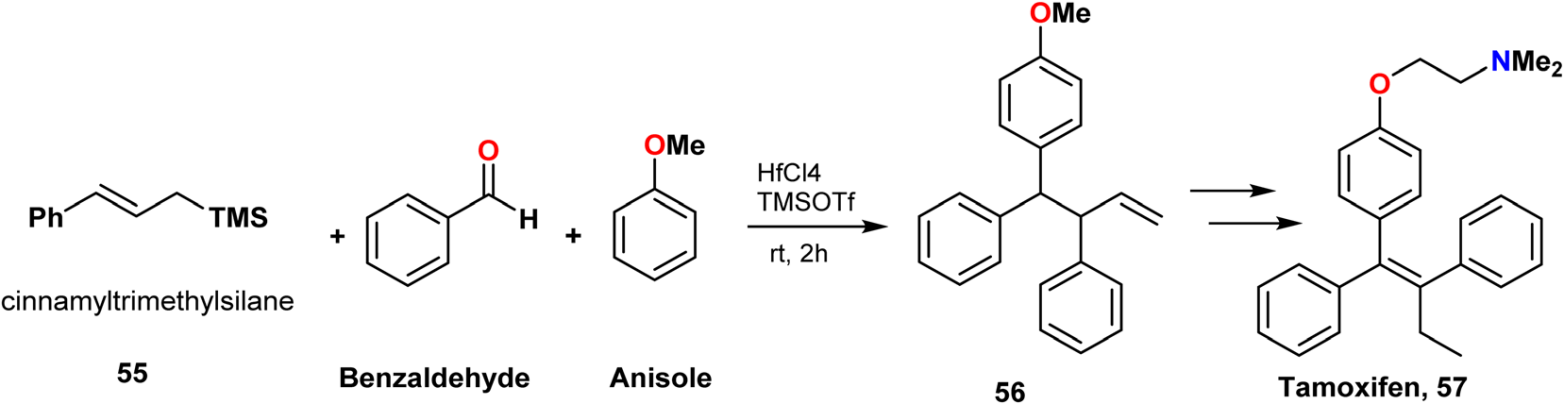
Synthesis of tamoxifen.

The reaction is typically promoted by a Lewis acid catalyst such as HfCl_4_ (hafnium tetrachloride) combined with trimethylsilyl trifluoromethanesulfonate. This coupling reaction yields 4-(4-methoxyphenyl)-3,4-diphenylbut-1-ene, 56 incorporating the anisole and benzaldehyde fragments into cinnamoyltrimethylsilane, 55. The intermediary but-1-ene, 56 is then subjected to successive double-bond migration to form the triarylethylene core. To obtain the final structure of tamoxifen, 57 the precursor undergoes further functionalization (*e.g.*, demethylation of anisole, alkylation with *N*,*N*-dimethylaminoethyl chloride) to introduce the characteristic basic side chain, (Fig. 11).

### 2.2 Favipiravir (T-705)

Due to its function as a broad-spectrum RNA-dependent RNA polymerase (RdRp) inhibitor, favipiravir (T-705), a fluorinated pyrazine carboxamide derivative that was once created for influenza, has been repurposed for the treatment of COVID-19. Its efficacy depends on the inclusion of a 6-fluoro group. In 2014, Toyama Chemical, a division of Fujifilm, authorized favipiravir (T-705) for medicinal use in Japan. In 2019, it was marketed under the brand name Avigan after going generic in 2016.^76^ In Japan, influenza is treated with this derivative of fluorinated pyrazine carboxamide.^77^ In contrast to other derivatives; T-705's pharmacokinetic character and biological efficacy were improved by the insertion of a 6-fluoro group at the pyrazine ring in the oral formulation.^78^

T-705 was repurposed for treating COVID-19 patients with mild to moderate illnesses exhibit clinical improvement and viral clearance.^79^

#### 2.2.1 Synthesis^80^

**Fig. 12:**
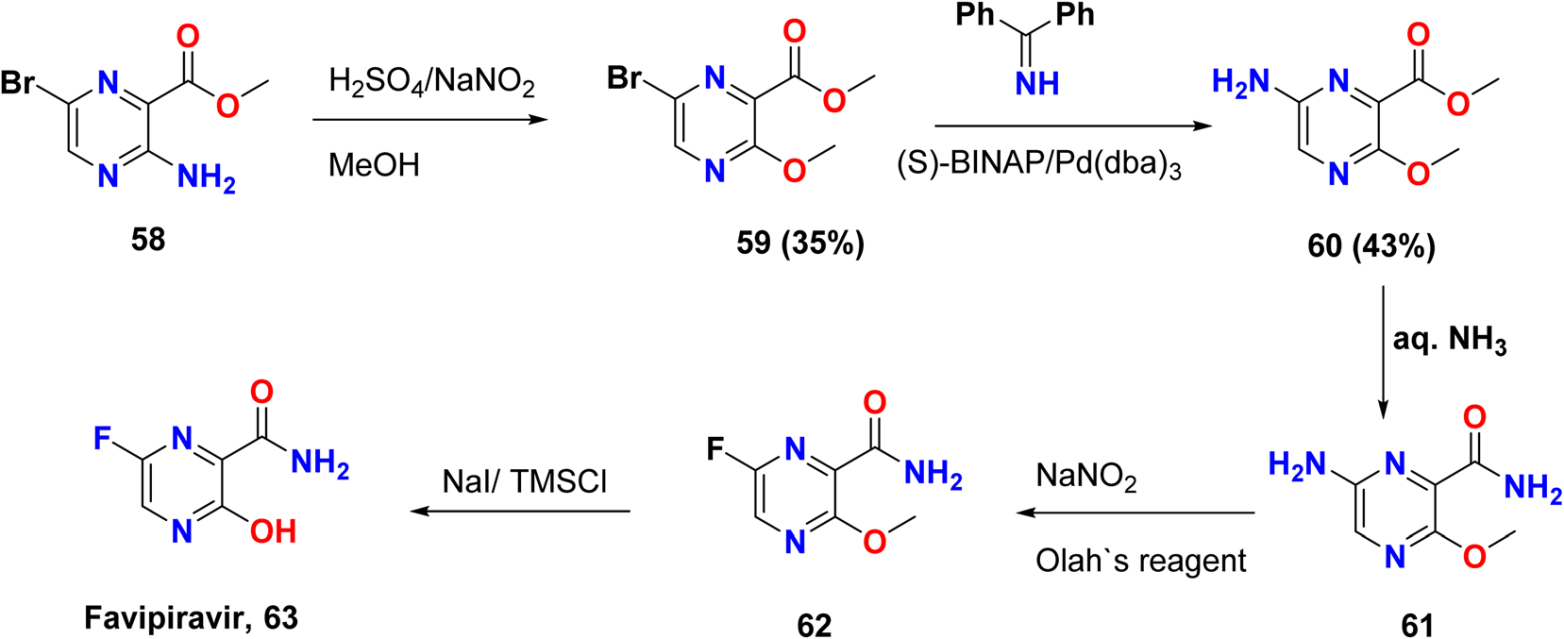
Synthesis of favipiravir.

Guo *et al.* synthesized favipiravir in 2019, as illustrated in Fig. 12. Beginning with pyrazine-2-carboxylate derivative 58, it was converted to its methoxy derivative, 59 by diazotization in methanol. After Buchwald–Hartwig amination of 59 produced 60, amidation with aqueous ammonia produced the equivalent amide 61. The fluorinated pyrazine derivative 62 was created by diazotizing 61 in the presence of pyridine hydrofluoride (Olah's reagent). The methyl ether 62 was then deprotected to produce favipiravir 63 as illustrated in Fig. 12.

### 2.3 Azidothymidine (AZT)

Nucleoside reverse transcriptase inhibitors (NRTIs) include azidothymidine (AZT) Gp C. was first studied as a chemotherapy treatment in 1964 *via* inhibiting telomerase activity. Azidothymidine (AZT) as well, enhancing paclitaxel-induced apoptosis in hypopharyngeal squamous cells, AZT. However, AZT failed to treat cancer in initial animal trials and was largely abandoned. It wasn't until the 1980s that researchers discovered its ability to inhibit retroviruses, leading to its historic repurposing as the very first medication used to treat HIV/AIDS.^81^

AZT's anti-retroviral effect was discovered early in the HIV epidemic in 1987, the FDA approved it as the first AIDS medication. The NIH collaborated with industry experts to repurpose the drug, which became the first treatment for HIV patients by acting as a viral DNA chain terminator and replacing thymidine in newly created viral DNA. This disrupts the HIV-1 life cycle by preventing HIV-1 reverse transcriptase from producing viral DNA from the RNA template and slows the progression to AIDS.^82^

#### 2.3.1 Synthesis^83^

**Fig. 13:**
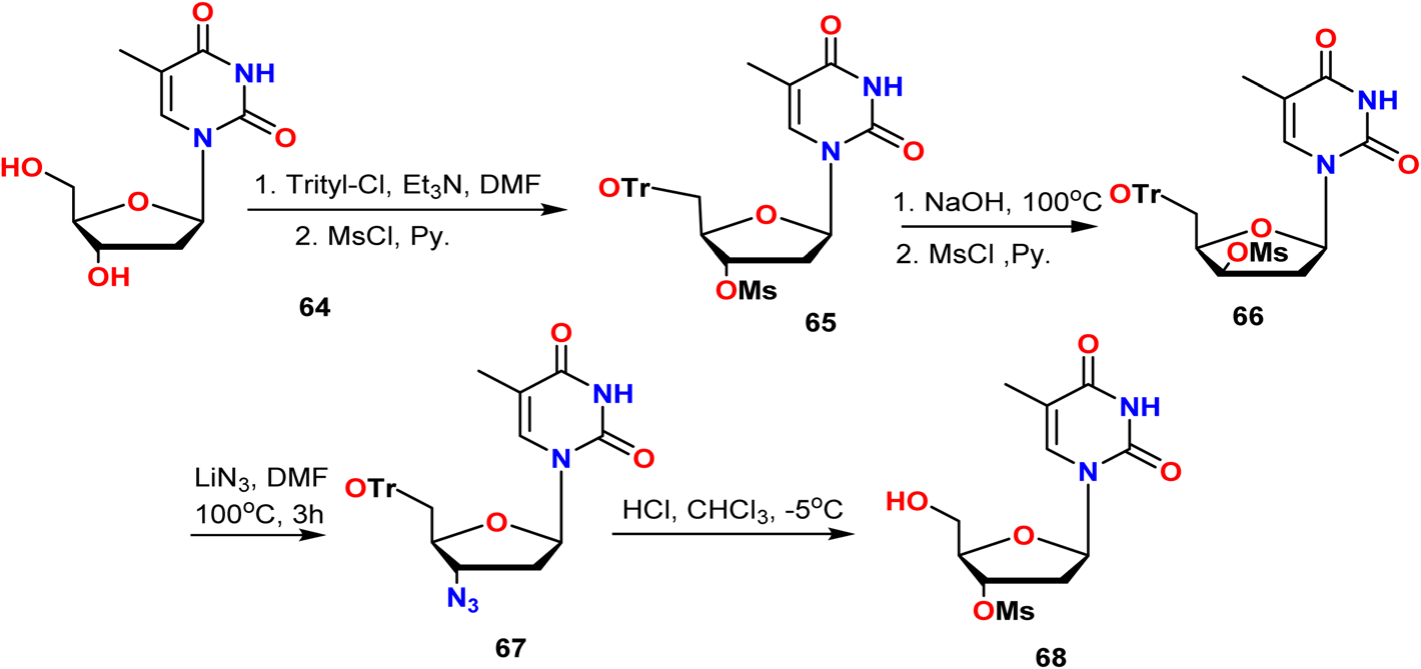
Synthesis of azidothymidine.

The 5′ hydroxyl of thymidine, 64 was protected by a trityl group, whilst the 3′ hydroxyl was activated as a methylate to synthesize compound 65 and hydrolysis created inverted stereochemistry at this position to yield 66. Then, the latter underwent nucleophilic displacement with lithium azide resulted in the azido substituent, 67. When the trityl group was deprotected in an acidic environment, AZT, 68 was created, (Fig. 13).

### 2.4 Sofosbuvir

(Sovaldi®, GS7977) 113 is primarily used to treat Hepatitis C approved by the FDA in 2013, manufactured by Gilead Sciences, Inc. It is a prodrug and has the advantage that the active phosphorylated nucleotide enhances both cell permeability and oral bioavailability. Its mechanism as an RNA-dependent RNA polymerase (RdRp) chain terminator targets the replication machinery shared by various pathogens.

Because SARS-CoV-2 shares significant structural and genetic homology with the Hepatitis C virus, researchers explored Sofosbuvir as a COVID-19 treatment. Sofosbuvir terminated RNA resists removal by the exonuclease to a substantially higher extent than RNA terminated by Remdesivir, another drug being used as a COVID-19 therapeutic. These results offer a molecular basis supporting the current use of Sofosbuvir in combination with other drugs in COVID-19 clinical trials.^84^

#### 2.4.1 Synthesis^85^

**Fig. 14:**
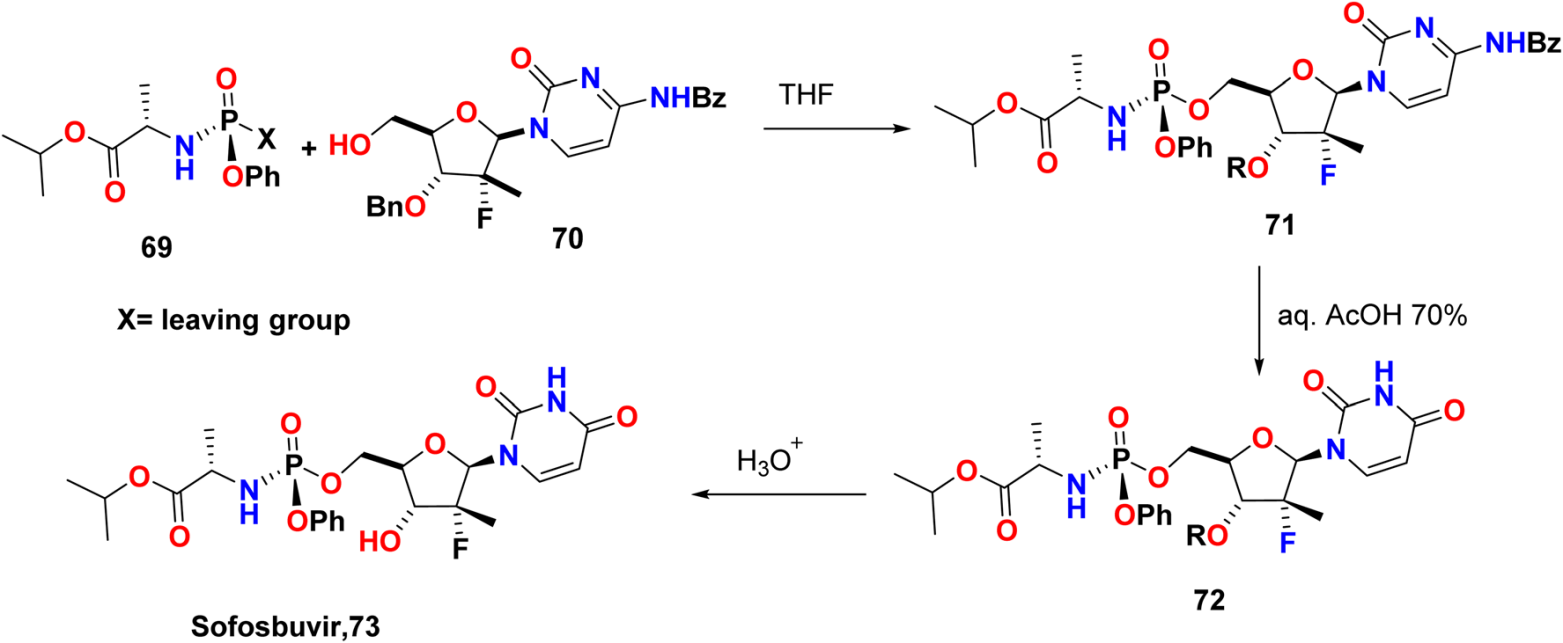
Synthesis of Sofosbuvir.

Its preparation was accomplished by Kaushik *et al.* in 2015 *via* the reaction of fluorinated deoxynucleoside 70 with the phosphonate 69 which contains a good leaving group (X) such as tosylate, camphorsulfonate, mesylate, trifluoroacetate, tri-fluorosulfonate, an aryloxide, or heteroaryl oxide substituted with at least one electron-withdrawing group to afford the key compound phosphonucleoside, 71. Then, debenzoylation was carried out *via* reaction of the latter with aqueous acetic acid 70% to produce 72. Subsequently, hydrolysis in the last step was performed using an aqueous acid such as hydrochloric acid, hydrobromic acid, sulfuric acid, formic acid, acetic acid, fumaric acid, oxalic acid, or mixtures thereof to provide sofosbuvir 73 Fig. 14.

### 2.5 Emtricitabine (FTC)

84 is a 5-fluorodeoxycytidine approved on 2003 and is involved the index of the important drugs assembled by the WHO.^86,87^ It is therapeutically employed for treatment of HIV infection in both adults and children *via* reverse transcriptase inhibition.^88,89^ U.S. Food and Drug Administration (FDA) at 2006 accepted the form of three-drug combination (Atripla), emtricitabine, tenofovir and efavirenz. Additionally, WHO registered two other combinations: emtricitabine/tenofovir and emtricitabine/efavirenz as indispensable drugs.

The repurposing of emtricitabine (typically in combination with tenofovir) for the treatment and prevention of COVID-19 gained significant scientific attention in 2020. A key observational study reported that individuals living with HIV who were receiving a combination of tenofovir disoproxil fumarate (TDF) and emtricitabine (FTC) had a lower risk of COVID-19 hospitalization compared to those on other therapies. Results from proof-of-concept trials were published in mid-2021, suggesting that the treatment might accelerate the natural clearance of the virus in outpatients.^90^

#### 2.5.1 Synthesis^91^

**Fig. 15:**
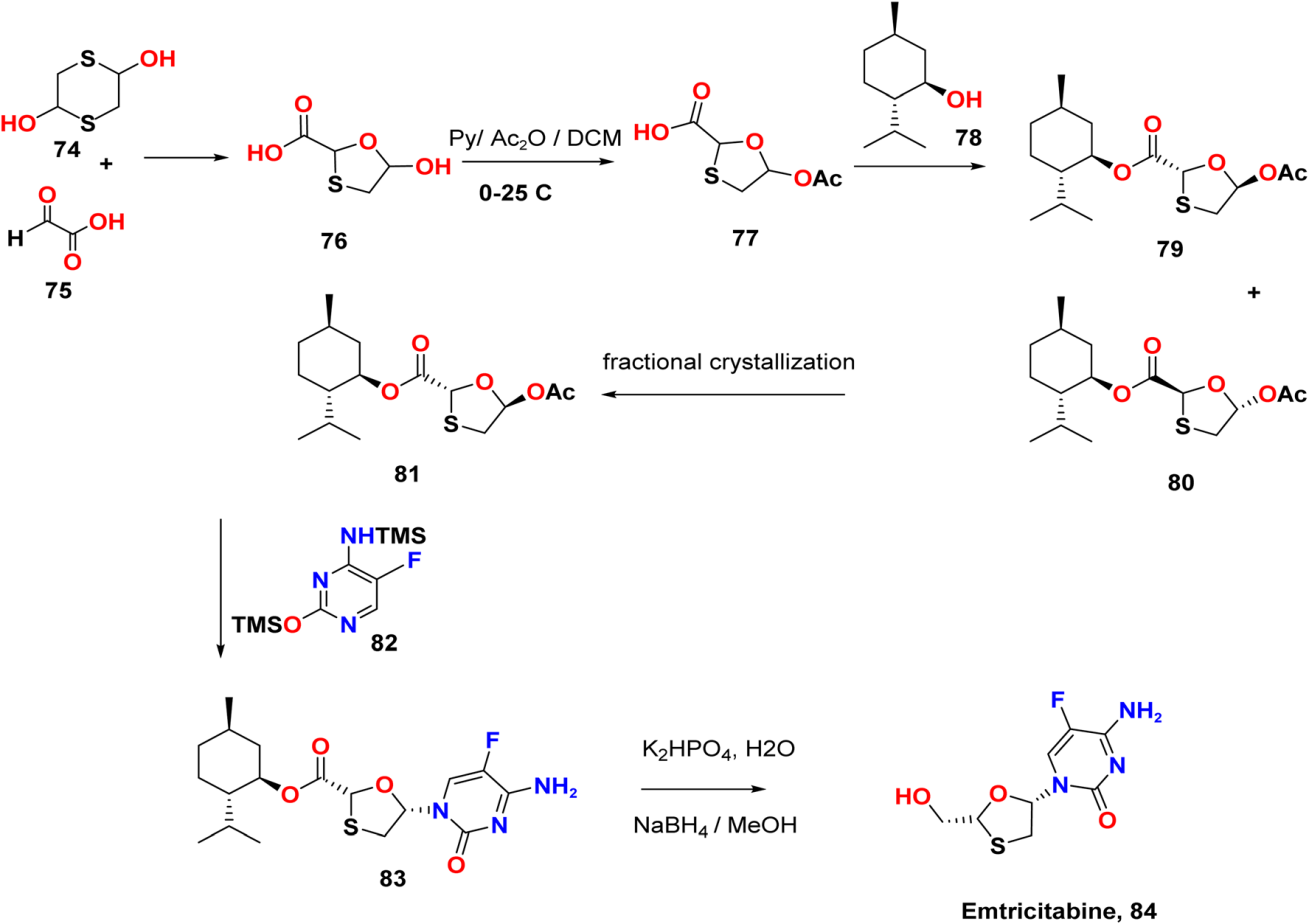
Synthesis of Emtricitabine.

Stereoselective synthetic route of emtricitabine 84 was accomplished starting from formation of *trans*-oxathiolane derivatives 76 from reaction of 2,5-dihydroxy-1,4-dithiane 74 with glyoxalic acid 75. Acetylation of 76 afforded 77 followed by esterification with a chiral auxiliary's 1-menthol 78 which led to a mixture of diastereoisomers 79 and 80. The desired diastereoisomer 81 was isolated by fractional crystallization and directly coupled with silylated 5-fluorocytosine 82, leading to the *N*-nucleoside derivative 83 which, finally, by reductive removal of the chiral auxiliary gives emitricitabine 84 represented with inversion of configuration in position 5 of oxathiolane,^91^Fig. 15.

### 2.6 Computational approaches and key structural features of repurposed medications

For every medication included in the review, Table 1 lists the original and repurposed indications, important structural factors related to repositioning, computational techniques used, primary molecular targets, and mechanistic insights of the mentioned FDA-approved medications are provided to support the review's medicinal chemistry perspectives.

**Table 1:** Computational approaches and structural determinants supporting drug repositioning of FDA-approved drugs

Drug	Original indication	Repurposed indication	Key structural feature associated with repositioning	Computational approach	Major target(s) investigated	Mechanistic insight
Aspirin	Analgesic/anti-inflammatory	Cancer chemoprevention	Acetylated salicylate moiety	Docking, pathway analysis^92^	COX-1, COX-2, NF-κB pathways	Reactive acetyl group permits covalent protein modification beyond cyclooxygenases
Thalidomide	Sedative	Multiple myeloma	Glutarimide–phthalimide scaffold	Structural biology, docking^93^	Cereblon (CRBN)	Glutarimide ring mediates cereblon binding and immunomodulatory activity
Vitamin C (ascorbic acid)	Nutritional supplement	Cancer supportive therapy	Enediol-lactone redox system	Network pharmacology^94^	Oxidative stress pathways	Redox-active structure regulates multiple cellular signaling processes
Metformin	Type 2 diabetes	Cancer and aging-related disorders	Biguanide moiety	Network pharmacology^95^	AMPK, mTOR, PI3K/Akt	Nitrogen-rich scaffold promotes energy-sensing pathway modulation
Niclosamide	Anthelmintic	Anticancer agent	Salicylanilide scaffold	Docking, network pharmacology^96^	Wnt/β-catenin, STAT3, NF-κB, mTOR	Polypharmacological scaffold interacts with multiple cancer-associated pathways
Gemcitabine	Antiviral investigations	Anticancer agent	Difluorinated nucleoside analogue	Docking, molecular modeling^97^	DNA/RNA polymerases	Fluorinated nucleoside scaffold disrupts nucleic acid synthesis in diverse systems
Orlistat	Obesity	Anticancer agent	β-Lactone-containing lipase inhibitor	Docking^98^	Fatty acid synthase (FASN)	Covalent enzyme inhibition extends beyond lipases to cancer-associated metabolic enzymes
Lidocaine	Local anesthetic	Anticancer adjunct therapy	Amino-amide pharmacophore	Docking^99^	Voltage-gated sodium channels	Membrane-active scaffold influences signaling pathways involved in metastasis and proliferation
Losartan	Hypertension	Solid tumors, fibrosis and inflammatory disorders	Biphenyl-tetrazole pharmacophore	Docking, network pharmacology^100^	AT1 receptor, TGF-β signaling	Conserved receptor-binding pharmacophore enables pathway modulation beyond blood pressure regulation
Atorvastatin	Hyperlipidemia	Anticancer and anti-inflammatory applications	Dihydroxyheptanoic acid pharmacophore	Network pharmacology^101,102^	HMG-CoA reductase, inflammatory pathways	Mevalonate pathway inhibition influences proliferation and immune regulation
Tamoxifen	Breast cancer	Antimicrobial and other non-oncologic uses	Triphenylethylene scaffold	Pharmacophore modeling, target prediction^103^	Estrogen receptor and secondary targets	Highly hydrophobic aromatic scaffold enables interaction with multiple proteins
Favipiravir	Influenza	COVID-19 and emerging RNA viral infections	Fluoropyrazine carboxamide scaffold	Docking, molecular dynamics^104^	Viral RNA-dependent RNA polymerase (RdRp)	Structural mimicry of nucleotide substrates facilitates viral polymerase inhibition
AZT (zidovudine)	Anticancer candidate	HIV/AIDS	Azido-substituted nucleoside analogue	Molecular modeling^105^	HIV reverse transcriptase	Modified nucleoside structure causes viral DNA chain termination
Sofobuvir	Hepatitis C	COVID-19	Uracil base	Molecular docking^106^	RNA-dependent RNA polymerase (RdRp)	Attaches selectively to viral polymerase protein and inhibits viral replication
			2′-Fluoro substituent			
			2′-*C*-methyl group	Knowledge-based repositioning^107^		
			2′-deoxy sugar			
Emtricitabine	HIV	COVID-19	Cytosine base analog	Structure-based approaches and AI-based computational drug design^108^	Nucleoside reverse transcriptase inhibitor (NRTI)	
			Oxathiolane ring			
			Nucleoside analog			

## 3 Conclusion and summary

In conclusion, current drug discovery has sparked interest in rapid drug development due to ever-expanding databases of compounds and therapeutic candidates. Drug repurposing is a very effective, cost-effective, and speedier alternative to traditional drug development, especially for rare or complex illnesses. It involves using currently available, safety-tested medications for novel therapeutic indications. This strategy is essential for treating uncommon diseases where new, conventional drug development is not financially feasible as well as pandemic crises *e.g.*, COVID-19 treatment. Notwithstanding its advantages, there are obstacles like patent problems, overcoming legal restrictions, and making sure additional indications undergo the same thorough efficacy testing. With AI and computational techniques speeding up the discovery of new applications, it is increasingly acknowledged as a long-lasting, essential part of pharmaceutical innovation rather than only a coincidental discovery. More collaboration between academia, industry, and government agencies is needed to fully exploit its potential, particularly in the battle against age-related illnesses.

## Author contributions

Magda M. F. Ismail (corresponding author)-conceived and designed the analysis and wrote the article, Taghreed Z. Shawer revised the manuscript, Omneya M. ElBakry-completed the synthetic procedures, Eman A. A. Baumy-contributed in collected the data, and checked the references and Sally I. Eissa revised the manuscript.

## Conflicts of interest

The authors declare that they have no conflicts of interest. The authors alone are responsible for the content and writing of this manuscript.

## Data Availability

All data is available within the published article and its cited references.
